# Self‐reported health, healthcare service use and health‐related needs: A comparison of older and younger homeless people

**DOI:** 10.1111/hsc.12739

**Published:** 2019-04-25

**Authors:** Sophie I. van Dongen, Barbara van Straaten, Judith R. L. M. Wolf, Bregje D. Onwuteaka‐Philipsen, Agnes van der Heide, Judith A. C. Rietjens, Dike van de Mheen

**Affiliations:** ^1^ Department of Public Health Erasmus University Medical Centre Rotterdam the Netherlands; ^2^ IVO Addiction Research Institute The Hague the Netherlands; ^3^ Radboud Institute for Health Sciences, Impuls ‐ Netherlands Centre for Social Care Research Radboud University Medical Centre Nijmegen the Netherlands; ^4^ Department of Public and Occupational Health, Expertise Centre for Palliative Care VU University Medical Centre, Amsterdam Public Health Research Institute Amsterdam the Netherlands; ^5^ School of Social and Behavioural Sciences, Tranzo Scientific Centre for Care and Welfare Tilburg University Tilburg the Netherlands

**Keywords:** care needs, homelessness, older people, perceived health, service use, social support

## Abstract

The number of older homeless people with a limited life expectancy is increasing. European studies on their health‐related characteristics are lacking. This study compared self‐reported health, healthcare service use and health‐related needs of older and younger homeless people in the Netherlands. It is part of a cohort study that followed 513 homeless people in the four major Dutch cities for a period of 2.5 years, starting from the moment they registered at the social relief system in 2011. Using cross‐sectional data from 378 participants who completed 2.5‐year follow‐up, we analysed differences in self‐reported health, healthcare service use, and health‐related needs between homeless adults aged ≥50 years (*N* = 97) and <50 years (*N* = 281) by means of logistic regression. Results show that statistically significantly more older than younger homeless people reported cardiovascular diseases (23.7% versus 10.3%), visual problems (26.8% versus 14.6%), limited social support from family (33.0% versus 19.6%) and friends or acquaintances (27.8% versus 14.6%), and medical hospital care use in the past year (50.5% versus 34.5%). Older homeless people statistically significantly less often reported cannabis (12.4% versus 45.2%) and excessive alcohol (16.5% versus 27.0%) use in the past month and dental (20.6% versus 46.6%) and mental (16.5% versus 25.6%) healthcare use in the past year. In both age groups, few people reported unmet health‐related needs. In conclusion, compared to younger homeless adults, older homeless adults report fewer substance use problems, but a similar number of dental and mental problems, and more physical and social problems. The multiple health problems experienced by both age groups are not always expressed as needs or addressed by healthcare services. Older homeless people seem to use more medical hospital care and less non‐acute, preventive healthcare than younger homeless people. This vulnerable group might benefit from shelter‐based or community outreach programmes that proactively provide multidisciplinary healthcare services.

**What is known about this topic**

- Compared to the general population, homeless people have more health problems and a substantially reduced life expectancy.
- They are considered old at the age of 50 years.
- During the past decade, the proportion of older homeless people increased.

**What this paper adds**

- Compared to younger homeless adults, older homeless adults have fewer substance use problems, but a similar number of dental and mental problems and more physical and social problems.
- Older homeless adults report more use of medical hospital care, but less use of dental and mental healthcare than younger homeless adults.
- Despite multiple health problems that are not always addressed by healthcare services, both older and younger homeless adults express few unmet health‐related needs.

## 1 INTRODUCTION

Compared to the general population, both formerly and currently homeless people have disproportionally high rates of physical and mental disorders, psychosocial problems, substance use disorders and multimorbidity (Fazel, Geddes, & Kushel, 2014; Nielsen, Hjorthoj, Erlangsen, & Nordentoft, 2011; Oppenheimer, Nurius, & Green, 2016). Additionally, they face specific challenges and barriers to accessing healthcare, often resulting in high rates of acute care use (Fazel et al., 2014). Furthermore, homelessness has shown to be an independent risk factor for mortality, with average ages of mortality varying roughly between 50 and 65 years in different studies in Western, high‐income countries (Baggett et al., 2013; Henwood, Byrne, & Scriber, 2015; Morrison, 2009; Nielsen et al., 2011; Stenius‐Ayoade et al., 2017). Therefore, at these generally still considered middle ages, homeless people are already classified as old (Fazel et al., 2014). Recent studies have demonstrated a large increase in the proportion of older homeless persons (Fazel et al., 2014; Hahn, Kushel, Bangsberg, Riley, & Moss, 2006). A similar demographic trend has been observed in the Netherlands: among homeless people receiving help from Dutch social relief facilities, the proportion of people older than 50 gradually increased, from 19% in 2009 to 26% in 2015 (Federation of Shelters, 2015). While the excess mortality among younger homeless people mainly results from external causes, such as injury and poisoning, older homeless people mainly die from chronic medical conditions, such as respiratory and cardiovascular diseases (Baggett et al., 2013; Stenius‐Ayoade et al., 2017). These findings suggest that differences between older and younger homeless people might also be present for other clinically relevant health outcomes. Nevertheless, only few studies have specifically examined health‐related characteristics of older homeless people (Brown et al., 2017; Brown, Kiely, Bharel, & Mitchell, 2012; Landefeld et al., 2017) or investigated health differences between older and younger homeless people (Abdul‐Hamid, 1997; Brown, Kimes, Guzman, & Kushel, 2010; Brown & Steinman, 2013; DeMallie, North, & Smith, 1997; Garibaldi, Conde‐Martel, & O'Toole, 2005; Gelberg, Linn, & Mayer‐Oakes, 1990; Hategan, Tisi, Abdurrahman, & Bourgeois, 2016; Kellogg & Horn, 2012; Kimbler, DeWees, & Harris, 2017; Nakonezny & Ojeda, 2005; Tompsett, Fowler, & Toro, 2009). The studies that have made such age comparisons consistently reported poorer physical health among older than younger homeless adults, but yielded conflicting results with regard to mental and psychosocial health, substance use, healthcare service use and health‐related needs (Abdul‐Hamid, 1997; Brown et al., 2010; Brown & Steinman, 2013; DeMallie et al., 1997; Garibaldi et al., 2005; Gelberg et al., 1990; Hategan et al., 2016; Kellogg & Horn, 2012; Kimbler et al., 2017; Nakonezny & Ojeda, 2005; Tompsett et al., 2009). Moreover, none of these studies have been conducted outside Canada and the United States, where healthcare and social relief systems are different than in Europe. In recent years, European organisations have increasingly advocated a life‐course approach to health, with more and more public health and social care policies being targeted at specific age groups (World Health Organisation Regional Office for Europe, 2015). Hence, identifying and understanding age‐related patterns in health of homeless populations is important, because this could contribute to more tailored healthcare provision for both older and younger vulnerable subgroups of homeless people. Therefore, the aim of this study was to describe self‐reported health, healthcare service use, and health‐related needs of older homeless adults (≥50 years) in the Netherlands and to compare these characteristics to those of younger Dutch homeless adults (<50 years).

## 2 METHODS

### 2.1 Ethics statement

This study complies with the criteria for studies that have to be reviewed by an accredited Medical Research Ethics Committee. Upon consultation, the Medical Research Ethics Committee region Arnhem–Nijmegen concluded that the study was exempt from formal review (registration number 2010/321). The study was conducted according to the principles formulated in the Code of Conduct for health research with data (http://www.federa.org). All participants were aged ≥18 years and provided written informed consent.

### 2.2 Design and study population

Data for this study were obtained from the CODA‐G4 study, an observational longitudinal cohort study that started data collection in 2011 and followed 513 homeless people in the four major cities in the Netherlands (i.e. Amsterdam, Rotterdam, The Hague and Utrecht) for a period of 2.5 years. It consisted of four measurement waves, that is, baseline, and six‐month, 1‐year, and 2.5‐year follow‐up. Procedures of sampling, data collection and response rates have been published previously (Van Straaten et al., 2016).

In this study, we included participants who completed all four waves. At baseline, all study participants satisfied the criteria set by the four major Dutch cities for registering at the social relief system and starting an individual programme plan. These included: aged ≥18 years, having legal status in the Netherlands, having stayed in the region of social relief application for at least two years during the last three years, having abandoned the home situation and not being sufficiently competent to live independently. In the Netherlands, registration at the social relief system is required to get access to social relief facilities (e.g. a night shelter). The individual programme plan was in 2006 implemented as part of the Strategy Plan for Social Relief (Dutch Government & four major cities, 2006), which aimed to provide homeless people in the four major Dutch cities with an income, suitable accommodation and effective support and to reduce the level of public nuisance caused by them. Most persons eligible for this study were literally homeless (e.g. sleeping in a night shelter or transitional accommodation, or staying temporarily with family, friends or acquaintances); a minority were either residentially homeless (e.g. residing in an institution) or housed but about to be evicted. At follow‐up, still being homeless was not required. Hence, the term “homeless” refers in this paper to people who had been homeless at baseline and were either still or formerly homeless at follow‐up.

### 2.3 Data collection and measurements

At each measurement, participants were interviewed face‐to‐face using a structured questionnaire (mean duration: 1.5 hr) administered by a trained interviewer at the participant's location of choice (generally a shelter facility, public library or the researcher's office). To enhance reliability of self‐reports, questions focused on recent events, interviewers did not wear any symbols of authority, participants’ privacy was ensured, and participants were stimulated to answer questions at their own pace (Gelberg & Siecke, 1997). All participants provided written informed consent. They received €15 for participation in the baseline interview, and €20, €25 and €30 for participation in the subsequent three follow‐up interviews, respectively.

All measures included in this study concern 2.5‐year follow‐up data (2014–2015), with the exception of suspected intellectual disability (see *Health‐related characteristics*), for which data had only been collected during the first, 6‐month follow‐up measurement (2011–2012).

#### 2.3.1 Background characteristics

We examined sociodemographic characteristics including sex, age, ethnicity, educational level, housing status, duration of homelessness, job status, health insurance status and living situation (Van Straaten et al., 2016; Wolf et al., 2002).

Age was calculated by subtracting participants’ date of birth from the date on which their 2.5‐year follow‐up measurement took place. In line with age‐specific public health and social policies (European Federation of National Organisations Working with the Homeless, 2009, 2011; European Parliamentary Research Services, 2014; European Social Network, 2008), we distinguished between older and younger participants: adopting the age cut‐off conventionally used in scientific literature about homeless populations (Fazel et al., 2014), participants aged ≥50 years were categorised into the older age group and participants aged <50 years (i.e. 18–49 years) were categorised into the younger age group. Using the ethnicity definition developed by Statistics Netherlands, participants were classified as “native Dutch” when they and both of their parents were born in the Netherlands, and as “having a foreign background” when they were foreign‐born or born in the Netherlands but with one or both of their parents being foreign‐born. Education was categorised as “low” when participants had completed pre‐vocational education, lower technical education, assistant training, basic labour‐oriented education or primary education at the most, and as “intermediate/high” when they had completed secondary vocational education, senior general secondary education, pre‐university education, higher professional education or university education. Housing status was measured by asking participants where they had slept last night. We categorised these locations into: (a) “homeless”: emergency shelter or night shelter; transitional accommodation (where the period of stay is intended to be short‐term); on the streets or in public spaces, (b) “institutionalised”: residential care or supported accommodation (long‐stay); medical institution, addiction care institution or psychiatric hospital; correctional or penal institution; residential care or supported accommodation, (c) “marginally housed”: staying with family, friends or acquaintances (temporarily), (d) “independently housed”: renting a house, room, or apartment, or owning one; residing with family, friends or acquaintances (permanently). Job status was assessed by asking participants whether or not they had a paid job or volunteer work in the year before the interview, a dichotomous item derived from The Dutch abbreviated version of the Lehman Quality of Life Interview (Wolf et al., 2002). The abbreviated Lehman Quality of Life interview was also used to assess on a dichotomous scale whether participants lived alone or with others (Wolf et al., 2002). Duration of homelessness upon admission to social relief was defined as the total number of months participants had been homeless ever in life. Health insurance status was measured by the yes‐or‐no question: “Do you have health insurance?”

#### 2.3.2 Health‐related characteristics

##### Health

Physical health was evaluated using an adapted version of the International Classification of Diseases (ICD‐10) questionnaire (World Health Organisation, 1994), comprising 14 of the original ICD‐10 categories, five additional categories of problems that are particularly common among homeless people (i.e. visual problems, auditory problems, dental problems, foot problems and fractures) (Levy & O'Connell, 2004) and a category “other.” We asked for the presence of problems in all categories over the last 30 days.

Mental health was examined using the concept of psychological distress. General psychological distress and symptoms of somatisation, depression and anxiety were measured using the Dutch translation of the Brief Symptom Inventory 18 (BSI‐18) (Derogatis, 2001). Based on comparisons with age‐specific (i.e. 18–29 years versus ≥30 years) and sex‐specific norm scores obtained in a general Dutch community sample (De Beurs, 2011), we classified symptom and general scores of the participants as “elevated” when they fell within the upper 20th percentile of the corresponding norm scores, and as “not elevated” when they fell outside of this range.

Suspected intellectual disability was measured using the Dutch version of the Hayes Ability Screening Index (HASI) (Hayes, 2000). HASI scores <85, corresponding to IQ scores <70 (Hayes, 2000), were considered to reflect suspected intellectual disability.

Substance use was assessed with a module of the European version of the Addiction Severity Index (EuropASI, version III) (Kokkevi, 1995). This module includes for various types of substances questions on whether they were used during the past month.

Social support was measured using five items derived from scales of the Medical Outcome Study (MOS) Social Support Survey (Sherbourne & Stewart, 1991). Participants were asked to indicate how often different kinds of support were available to them through (a) family and (b) friends or other acquaintances, on a five‐point scale ranging from “never” to “always”. By averaging across items and, subsequently, dichotomising the ordinal 0–5 scores, we constructed for both types of social support (i.e. (a) family and (b) friends or other acquaintances) a summary measure, reflecting the proportions of participants who rarely or never experienced support.

##### Healthcare service use

Healthcare (including social care) service use during the past year was examined with a questionnaire developed by the Netherlands Centre for Social Care Research (Impuls) (Lako et al., 2013). This questionnaire includes questions about the use of general practice (GP) care, medical hospital care, dental care, mental healthcare, addiction care and social work.

##### Unmet health‐related needs

Unmet health‐related needs were explored using a questionnaire developed by the Netherlands Centre for Social Care Research (Impuls) (Lako et al., 2013). For the present study, we considered needs in five domains: physical health, dental health, mental health, substance use and social relationships. For each domain, the following two questions were asked: “Do you want help with …?” and “Do you get help with…?” Subsequently, we created for each domain a dichotomous unmet need variable, which was scored affirmatively when participants indicated that they wanted, but did not receive help.

### 2.4 Data analyses

Potential differences in background characteristics between older and younger homeless participants were tested using independent *t *tests or non‐parametric Mann–Whitney *U* tests for continuous variables and Fisher's exact tests for dichotomous and polytomous categorical variables. Multiple logistic regression analyses were performed with each of the health‐related measures as dependent variable and age group (dichotomous; ≥50 years versus <50 years) as primary independent variable. Subsequently, we adjusted these crude models for background characteristics that were statistically significantly different between the age groups. In exploratory analyses, we additionally adjusted models examining associations between age group and healthcare service use for health problems that were univocally relatable to the healthcare service type of interest (e.g. additional adjustment for dental problems in the model examining the association between older age and dental care use). *p* Values <0.05 and 95% confidence intervals (95% CIs) not containing odds ratio (OR) = 1 were considered to indicate statistically significant differences.

## 3 RESULTS

### 3.1 Background characteristics

Of the initial cohort of 513 participants, 378 persons (i.e. 73.7% response) were interviewed again for the 2.5‐year follow‐up measurement. Of the 135 non‐respondents, 1 person died, 22 persons refused to participate, and 112 persons were lost to follow‐up. The percentages of attrition were not statistically significantly different between the older (20.0%) and the younger (28.0%) age groups (*p* = 0.077).

Our study sample consisted of 97 (25.7%) older participants (i.e. ≥50 years) and 281 (74.3%) younger participants (i.e. <50 years). Table 1 summarises the background characteristics of both age groups. Compared to younger participants, older participants were statistically significantly more often native Dutch (*p* < 0.001). The age groups were not different with regard to sex, educational level, job status, housing status, living situation, lifetime duration of homelessness and health insurance status (all *p* > 0.05). The majority of both older (59.8%) and younger (54.1%) participants had moved back into stable, independent housing at the time of the 2.5‐year follow‐up measurement.

**Table 1:** Background characteristics of older and younger homeless adults (*N* = 378)

Background characteristic	Older adults: ≥50 years (*N*_total_ = 97)	Younger adults: <50 years (*N*_total_ = 281)	*p* value^a^
	%/ Mean (*SD*)/ Median [IQR]	%/ Mean (*SD*)/ Median [IQR]	
Age in years	56.9 (5.5)	33.7 (8.8)	*<0.001*
Sex: male	78.4	72.2	0.284
Ethnicity: having a foreign background^b^	47.9	69.9	*<0.001*
Education: low^c^	56.5	66.8	0.098
Having had a job and/ or volunteer work in the past year	52.6	54.8	0.724
Housing status:			0.115
homeless	2.1	2.8	
institutionalised	35.1	32.4	
marginally housed	3.1	10.7	
independently housed	59.8	54.1	
Living situation: alone^d^	79.4	69.3	0.066
Lifetime duration of homelessness upon social relief admission^e^			
Number of months	11.0 [5.0–43.5]	12.0 [4.0–36.0]	0.737
≥1 year	49.5	53.9	0.480
Health insurance^f^	97.8	95.7	0.532

Note

*N*: number; *SD*: standard deviation; IQR: interquartile range.

a Continuous characteristics presented as mean (*SD*): Independent *t* test; Continuous characteristics presented as median [IQR]: Mann–Whitney *U* test; Categorical characteristics: Fisher's exact test; Statistically significant *p* values (*p* < 0.05) are printed in italics.

b Missing: ≥50 years: *N* = 1; <50 years: *N* = 9.

c Missing: ≥50 years: *N* = 5; <50 years: *N* = 34.

d Missing: ≥50 years: *N* = 0; <50 years: *N* = 1.

e Missing: ≥50 years: *N* = 0; <50 years: *N* = 1.

f Missing: ≥50 years: *N* = 4; <50 years: *N* = 3.

### 3.2 Health‐related characteristics

#### 3.2.1 Health

Results regarding health status of older versus younger participants are presented in Table 2. The most frequently mentioned physical problems (i.e. mentioned by ≥20.0% in one or both of the age groups) were musculoskeletal, respiratory, and cardiovascular diseases, and dental and visual problems. After adjustment for ethnicity (i.e. the only background characteristic for which the age groups statistically significantly differed), older participants reported more cardiovascular diseases and visual problems than younger participants (ORs [95% CIs] = 2.49 [1.33–4.67] and 2.17 [1.22–3.85], respectively).

**Table 2:** Health of older and younger homeless adults (*N* = 378)

Characteristic	Older adults: ≥50 years	Younger adults: <50 years	OR _(crude)_ a	95% CI	OR _(adjusted)_ a ^,^ b	95% CI
	(N _total_ = 97)	(N _total_ = 281)				
	%	%				
Physical problems (ICD‐10; 20 categories)						
Reported by ≥20.0% in ≥1 of the age groups:						
Musculoskeletal diseases	34.0	27.4	1.37	0.83–2.24	1.13	0.68–1.89
Respiratory diseases	25.8	17.4	1.64	0.95–2.85	1.43	0.81–2.53
Cardiovascular diseases	23.7	10.3	*2.70*	1.47–4.95	*2.49*	1.33–4.67
Dental problems	27.8	24.6	1.19	0.70–1.99	1.15	0.68–1.97
Visual problems	26.8	14.6	*2.14*	1.23–3.75	*2.17*	1.22–3.85
**At least one complaint**	78.4	67.3	*1.76*	1.02–3.03	1.61	0.93–2.81
Mental health problems (BSI‐18): elevated score^c^						
Anxiety	27.8	25.6	1.12	0.67–1.88	1.16	0.68–1.98
Depression	23.2	26.4	0.84	0.49–1.45	0.85	0.49–1.49
Somatisation	27.8	25.6	1.12	0.67–1.88	1.12	0.66–1.90
**Total psychological distress**	27.4	27.1	1.01	0.60–1.71	1.05	0.61–1.80
Suspected intellectual disability (HASI)^d^	33.7	29.5	1.21	0.72–2.05	1.17	0.68–2.01
Substance use in the past month (EuropASI‐III)						
Reported by ≥10.0% in ≥1 of the age groups:						
Tobacco	73.2	76.2	0.86	0.51–1.45	0.76	0.44–1.32
Analgesics^e^	29.9	22.8	1.45	0.86–2.42	1.40	0.82–2.38
Alcohol (≥5 units)	16.5	27.0	*0.53*	0.29–0.97	*0.44*	0.24–0.82
Cannabis^f^	12.4	45.2	0.17	0.09–0.33	*0.17*	0.09–0.33
Benzodiazepines^g^	11.3	13.9	0.79	0.39–1.62	0.75	0.36–1.55
Social support (MOS Social Support Survey)^h^						
Family: rarely or never	33.0	19.6	*2.02*	1.21–3.39	*1.80*	1.05–3.09
Friends/Acquaintances: rarely or never	27.8	14.6	*2.26*	1.30–3.93	*2.18*	1.23–3.87

Note

*N*: number; OR: odds ratio; CI: confidence interval; ICD‐10: International Classification of Diseases 10; BSI‐18: Brief Symptom Inventory 18; HASI: Hayes Ability Screening Index; EuropASI‐III European Addiction Severity Index version III; MOS: Medical Outcome Study.

a Statistically significant ORs (i.e. 95% CI not containing OR = 1; corresponding to *p* < 0.05) are printed in italics.

b Adjusted for ethnicity (foreign versus native Dutch background).

c Elevated BSI‐18 scores: scores within the upper 20th percentile of the corresponding norm scores obtained in a Dutch community sample; Missing depression symptom score: ≥50 years: *N* = 2; <50 years: *N* = 1; Missing total psychological distress score: ≥50 years: *N* = 2; <50 years: *N* = 1.

d Suspected intellectual disability: HASI score <85, corresponding to IQ <70; No suspected intellectual disability: HASI score ≥85, corresponding to IQ ≥70; Missing: ≥50 years: *N* = 8; <50 years: *N* = 54.

e Using analgesics without medical prescription: ≥50 years: 12.4%; <50 years: 17.1% (*p* = 0.334).

f Using cannabis without medical prescription: ≥50 years: 11.3%; <50 years: 44.1% (*p* < 0.001).

g Using benzodiazepines without medical prescription: ≥50 years: 2.1%; <50 years: 2.1% (p ≈ 1.000).

h Ordinal subscale scores ranging from 0 (“never”) to 4 (“always”); Category “rarely or never” constructed by combining categories 3 (“rarely”) and 4 (“never”).

Psychological distress was reported by about 27% in both age groups. Thirty‐four percent (33.7%) of the older and 29.5% of the younger participants had suspected intellectual disability; this difference was not statistically significant.

About three quarters of both older (73.2%) and younger (76.2%) participants reported smoking in the past month. Analgesics, alcohol (≥5 units per occasion), cannabis and benzodiazepines had also been used by ≥10.0% in one or both age groups, with statistically significantly less use of alcohol and cannabis by older compared to younger participants (ORs [95% CIs] = 0.44 [0.24–0.82] and 0.17 [0.09–0.33], respectively).

About twice as many older participants as younger participants rarely or never experienced social support from family (33.0% versus 19.6%; OR [95% CI] = 1.80 [1.05–3.09]) and friends or other acquaintances (27.8% versus 14.6%; OR [95% CI] = 2.18 [1.23–3.87]).

#### 3.2.2 Healthcare service use

Older participants reported more often than younger participants to have used medical hospital care in the past year (50.5% versus 34.5%; OR [95% CI] = 1.70 [1.05–2.76]; see Table 3). However, they less often reported use of dental (20.6% versus 46.6%; OR [95% CI] = 0.30 [0.17–0.52]) and mental healthcare (16.5% versus 25.6%; OR [95% CI] = 0.89 [0.52–1.42]) during this period.

**Table 3:** Self‐reported healthcare service use of older and younger homeless adults (*N* = 378)

Type of service use in the past year	Older adults: ≥50 years (*N*_total_ = 97)	Younger adults: <50 years (*N*_total_ = 281)	OR _(crude)_ a	95% CI	OR _(adjusted)_ a ^,^ b	95% CI
GP care	58.8	59.1	0.99	0.62–1.58	0.89	0.55–1.44
Medical hospital care	50.5	34.5	*1.94*	1.21–3.09	*1.70*	1.05–2.76
Dental care	20.6	46.6	*0.30*	0.17–0. 51	*0.30*	0.17–0.52
Mental healthcare	16.5	25.6	0.57	0.32–1.04	*0.54*	0.29–0.99
Addiction care	10.3	11.4	0.89	0.42–1.90	0.76	0.35–1.65
Social work	35.1	44.8	0.66	0.41–1.07	0.66	0.41–1.08

Note

*N*: number; OR: odds ratio; CI: confidence interval; GP: general practice.

a Statistically significant ORs (i.e. 95% CI not containing OR = 1; corresponding to *p* < 0.05) are printed in italics.

b ORs adjusted for ethnicity (foreign versus native Dutch background).

Exploratory analyses (results not shown in Table 3) indicated that greater use of medical hospital care among older compared to younger participants was largely explained by the higher prevalence of physical complaints among the older age group; after additional adjustment for physical complaints, the association between older age and medical hospital care use disappeared (OR [95% CI] = 1.61 [0.98–2.63]), whereas the association between physical complaints and medical hospital care use was highly statistically significant (OR [95% CI] = 2.17 [1.32–3.58]). Additional adjustment for total psychological distress did not explain why fewer older than younger participants reported mental healthcare use, as, irrespective of a statistically significant positive association between total psychological distress and mental healthcare use (OR [95% CI] = 3.68 [2.19–6.20]), the negative association between older age and mental healthcare use remained statistically significant (OR [95% CI] = 0.52 [0.27–0.98]). Additional adjustment for dental problems did not affect the finding that less older than younger participants used dental care (OR [95% CI] = 0.30 [0.17–0.52]); in this exploratory model, dental problems were not associated with dental care use (OR [95% CI] = 0.89 [0.52–1.42]).

#### 3.2.3 Unmet health‐related needs

Among both older and younger age groups, unmet health‐related needs were most prevalent in the domain of dental health (24.7% versus 31.3%; see Table 4). For each of the other health domains, unmet needs were reported by <15.0% of both older and younger age groups. For these domains, only few of the remaining older and younger participants reported to have needs that were met; nearly all of them indicated to have no needs at all (i.e. range older versus younger participants: 70.5% versus 77.5% (physical health) — 100.0% versus 98.9% (substance use); results not shown in Table 4).

**Table 4:** Self‐reported unmet health‐related needs of older and younger homeless adults (*N* = 378)

	Older adults: ≥50 years	Younger adults: <50 years				
	(N _total_ = 97)	(N _total _ =281)				
Domain	%	%	OR _(crude)_ a	95% CI	OR _(adjusted)_ a, b	95% CI
Physical health^c^	9.3	14.0	0.63	0.29–1.35	0.65	0.30–1.42
Dental health	24.7	31.3	0.72	0.43–1.22	0.77	0.45–1.33
Mental health^d^	5.2	10.0	0.49	0.18–1.30	0.33	0.20–1.43
Substance use^e^	1.1	6.2	0.17	0.02–1.28	0.18	0.02–1.40
Use of alcohol^f^	1.1	2.9	0.37	0.05–2.98	0.36	0.04–2.96
Use of drugs^g^	0.0	5.1	0.00	—	0.00	—
Social relationships^h^	3.1	2.8	1.10	0.29–4.24	0.98	0.25–3.89
Relationship with family^i^	7.3	4.0	1.90	0.71–5.04	1.97	0.72–5.35
Relationship with own child(ren)^j^	0.0	9.0	0.00	—	0.00	—

Note

*N*: number; OR: odds ratio; CI: confidence interval.

a Statistically significant ORs (i.e. 95% CI not containing OR = 1; corresponding to *p* < 0.05) are printed in italics.

b ORs adjusted for ethnicity (foreign versus native Dutch background).

c Missing: ≥50 years: *N* = 0; <50 years: *N* = 2.

d Missing ≥50 years: *N* = 0; <50 years: *N* = 2.

e Missing: ≥50 years: *N* = 6; <50 years: *N* = 7.

f Missing: ≥50 years: *N* = 5; <50 years: *N* = 5.

g Missing: ≥50 years: *N* = 6; <50 years: *N* = 4.

h Missing: ≥50 years: *N* = 1; <50 years: *N* = 0.

i Missing: ≥50 years: *N* = 1; <50 years: *N* = 5.

j No children: ≥50 years: *N* = 28; <50 years: *N* = 153; Missing: ≥50 years: *N* = 2; <50 years: *N* = 6.

## 4 DISCUSSION

### 4.1 Summary of findings

We compared health‐related characteristics between older and younger homeless adults in the four major Dutch cities and showed that, compared to younger homeless adults, older homeless adults more often reported cardiovascular diseases, visual problems, limited social support, and medical hospital use during the past year. Conversely, they less often reported cannabis and excessive alcohol use in the past month and dental and mental healthcare use in the past year. Both older and younger homeless people experienced multiple health problems, but, especially among older homeless people, not all these problems were addressed by healthcare services. Nevertheless, in both age groups, few people expressed unmet health‐related needs.

Consistent with results of previous studies among homeless people, we found that older age was associated with worse physical health (Abdul‐Hamid, 1997; Brown & Steinman, 2013; Garibaldi et al., 2005; Gelberg et al., 1990; Kellogg & Horn, 2012; Kimbler et al., 2017; Nakonezny & Ojeda, 2005; Tompsett et al., 2009) and that about 30 percent of both the older and younger homeless people had elevated levels of psychological distress (DeMallie et al., 1997; Garibaldi et al., 2005) and were screened positive for suspected intellectual disability (Hurstak et al., 2017; Spence, Stevens, & Parks, 2004). Among both older and younger homeless people in our study, levels of substance use were lower than generally reported in studies among homeless populations (DeMallie et al., 1997; Garibaldi et al., 2005; Landefeld et al., 2017; Nielsen et al., 2011; Tompsett et al., 2009). As many as 33.0% and 27.8% of the older participants, compared to 19.6% and 14.6% of the younger participants in our study reported to only rarely or never receive social support from family and friends or acquaintances, respectively. This finding warrants attention, all the more because several studies in other Western, high‐income countries did comparable observations, showing that older homeless people had fewer informal social contacts (Gelberg et al., 1990) and smaller social networks (Tompsett et al., 2009), and stayed in shelters that were further removed from their emergency contacts (Kimbler et al., 2017) than younger homeless people. Moreover, lack of social support has found to be predictive of new episodes of homelessness (Duchesne & Rothwell, 2016).

We found several differences in healthcare service use between older and younger participants. Compared to younger participants, older participants reported more use of medical hospital care in the past year. Remarkably, despite reporting similar and even higher levels of dental and mental health problems, they reported relatively less use of dental and mental healthcare services, respectively. Also in exploratory statistical models, these age differences in healthcare service use could not be explained by the prevalence of corresponding health problems. Indirectly, a comparable association was observed between social support and use of social and mental healthcare services: while older homeless participants reported more social problems, they did not use more social or mental healthcare services than younger homeless participants. These distinct age‐related patterns of healthcare service use may thus indicate that older homeless people are less inclined than younger homeless people to use non‐acute and/ or preventive care. This interpretation is supported by an American study that showed that older homeless people, while suffering from more diseases, were less likely to seek out‐of‐hospital care than younger homeless people (Gelberg, Andersen, & Leake, 2000).

Despite multiple health problems, both older and younger homeless participants in this study expressed few unmet health‐related needs. Furthermore, rather than reporting that their needs had been met, in most health domains, the vast majority of participants reported no needs at all. These results are noteworthy, as they do not seem to match the finding that health problems of especially the older participants were not always addressed by healthcare services.

Our study thus suggests that among homeless people, there is a mismatch between health problems on the one hand, and healthcare service use and expressed health‐related needs on the other hand. This mismatch seemed to be more pronounced among older participants, who reported more health problems but less use of non‐acute, preventive healthcare services and (slightly) fewer health‐related needs than younger participants. Possibly, having become used to certain health problems or perceiving them as untreatable or normal for their age interfered with expressing needs and seeking healthcare services among especially the older participants in our study. The observed discrepancies might also be explained by competing priorities and hierarchy of needs in multiple care domains. Indeed, in the CODA‐G4 cohort, as well as in two other studies assessing needs among older and younger homeless people, participants reported considerably more needs in domains of housing and residential care, finance and/ or work than in domains of health (Abdul‐Hamid, 1997; Garibaldi et al., 2005; Van Straaten et al., 2017). Also, homeless people have been shown to make less use of non‐acute medical services as levels of competing subsistence priorities increase (Gelberg, Gallagher, Andersen, & Koegel, 1997).

### 4.2 Strengths and limitations

To our knowledge, this is the first European study to assess and compare self‐reported health, healthcare service use and health‐related needs of older and younger homeless adults. Using a broad and pragmatic definition of homelessness enabled us to reach a substantial, heterogeneous part of the urban Dutch homeless population. However, because all participants were recruited upon admission to the social relief system in 2011, many of them were newly homeless; more than half of them had a lifetime duration of homelessness of less than one year. Since several studies in Western, high‐income countries have shown that health outcomes deteriorate as the duration of homelessness increases (Fazel et al., 2014), potential underrepresentation of chronically homeless people may have resulted in an underestimation of the health problems in the total homeless population. Additionally, so‐called “hidden” minority subgroups of undocumented homeless people and homeless people who do not use social relief facilities were not included in our study, thus limiting generalisability of study results to these subgroups. Furthermore, although we included comprehensive measures in multiple domains of health, we unfortunately did not have data to examine all relevant health‐related characteristics, such as psychotic disorders and activities of daily living. Finally, most of our data concerned self‐reports, which are often assumed to be more biased by reporting errors and social desirability than for example register‐based data. Yet, homeless people have been shown to be quite accurate reporters of health and healthcare experiences (Gelberg & Siecke, 1997; Hwang, Chambers, & Katic, 2016) and, moreover, we applied several methods to enhance self‐report reliability (Gelberg & Siecke, 1997). Also, while previous self‐report studies often used quick, single‐item assessments, all questionnaires administered in this study consist of multiple items and have been validated in homeless or other socially vulnerable populations (Van Straaten, 2016). Most importantly, gaining insight into the perceptions of homeless people themselves is crucial for developing tailored and effective health interventions (Manary, Boulding, Staelin, & Glickman, 2013).

### 4.3 Conclusion and policy implications

This study shows that, compared to younger homeless adults, older homeless adults experience lower levels of substance use, yet considerably higher levels of physical and social problems, and similarly high levels of dental and mental health problems. Their healthcare service use seems to involve considerably more medical hospital care and less non‐acute, preventive care. Despite multiple health problems that are not always addressed by healthcare services, both older and younger homeless adults express few unmet health‐related needs.

Our findings illustrate the complexity of adequately and efficiently providing healthcare to this socially marginalised population. Older homeless people, who have multiple health problems but often lack social support and do not explicitly express health‐related needs, appear to be even more dependent on proactively provided professional healthcare than younger homeless people. Thus far, however, they have typically fallen outside the scope of European health and social care policies directed at specific age groups, for example, homeless youth and (frail) people aged over 65 (European Federation of National Organisations Working with the Homeless, 2009, 2011; European Parliamentary Research Services, 2014; European Social Network, 2008). They might benefit from shelter‐based and community outreach programmes that provide multidisciplinary healthcare services including various types of non‐acute and/ or preventive care.

## CONFLICTS OF INTEREST

No conflicts of interest have been declared.
